# Continuous Enhancement of Science Teachers’ Knowledge and Skills through Scientific Lecturing

**DOI:** 10.3389/fpubh.2018.00041

**Published:** 2018-02-27

**Authors:** Maria-Manuel Azevedo, Sofia Duarte

**Affiliations:** ^1^Agrupamento de Escolas D. Maria II, Vila Nova de Famalicão, Portugal; ^2^Department of Microbiology, Faculty of Medicine, University of Porto, Porto, Portugal; ^3^Center for Research in Health Technologies and Information Systems, Faculty of Medicine, University of Porto, Porto, Portugal; ^4^Centre of Molecular and Environmental Biology (CBMA), Department of Biology, University of Minho, Braga, Portugal

**Keywords:** adult continuing education, teacher training in sciences, scientific lecturing, environmental education, health education

## Abstract

**Introduction:**

Due to their importance in transmitting knowledge, teachers can play a crucial role in students’ scientific literacy acquisition and motivation to respond to ongoing and future economic and societal challenges. However, to conduct this task effectively, teachers need to continuously improve their knowledge, and for that, a periodic update is mandatory for actualization of scientific knowledge and skills. This work is based on the outcomes of an educational study implemented with science teachers from Portuguese Basic and Secondary schools. We evaluated the effectiveness of a training activity consisting of lectures covering environmental and health sciences conducted by scientists/academic teachers.

**Material and methods:**

The outcomes of this educational study were evaluated using a survey with several questions about environmental and health scientific topics. Responses to the survey were analyzed before and after the implementation of the scientific lectures.

**Results:**

Our results showed that Basic and Secondary schools teachers’ knowledge was greatly improved after the lectures. The teachers under training felt that these scientific lectures have positively impacted their current knowledge and awareness on several up-to-date scientific topics, as well as their teaching methods.

**Learning outcomes:**

This study emphasizes the importance of continuing teacher education concerning knowledge and awareness about health and environmental education.

## Introduction

Scientific education plays a crucial role in human development and in the acquisition of endogenous scientific capacity, a decisive step toward the development of skills for intervenient and informed citizens (1). In this sense, scientific literacy is of high priority for all and may help citizens to be interested in several environmental and societal challenges and in making informed decisions about their own health and well-being (2). In addition, lifelong learning has been shown to positively impact adults’ knowledge and principles and to conduct to more prolific life experiences [American association for adult and continuing education (AAACE)].^1^ Therefore, the communication of science to the general public can play a major role in the twenty-first century society, as highlighted in the action plan “Science and Society,” designed by the European Commission (3). In addition, there is an increasing concern within the scientific community in organizing teams to prepare press releases and communicate science that build the public awareness to scientific research and its benefits to tackle societal challenges (3–5). Academic teachers and scientists are at the front line in providing scientific literacy to societies, such as in terms of health promotion (6, 7) and environmental protection (8). Previous evidence suggested that teachers significantly enhanced skills in communicating science to students, after their involvement in research programs (hands-on practice science) (9).

In Basic and Secondary schools, teachers can play a crucial role in providing scientific literacy to their students, although, nowadays, young students have access to many sources of information (e.g., science-related TV/radio programs). However, to conduct this task effectively, teachers need to continuously improve their knowledge, and for that, a periodic update is mandatory for actualization of scientific knowledge and skills (9). However, to increase students’ perception of science, it is very important to make a connection between theoretical knowledge and practical examples, in the light of the most recent scientific advances. Continuous teachers training involving all important players of communication of science, such as the media, researchers, research institutions, universities, and business companies may help to fulfill these gaps (10). The need to update teacher competencies has its origins in the middle of the twentieth century, when competency-based teacher education became common (11). However, after some decades, a more humanistic approach was adopted to highlight the need to focus on the process of becoming a teacher, on the teacher as a person and not only on the lists of skills (11, 12).

Several interesting partnership programs between universities and schools have been established [e.g., University of California, San Francisco (UCSF)—and San Francisco Unified School District (13); Portuguese universities and Basic schools or secondary students, teachers and parents, such as Junior University program, by University of Porto; Living Science or Hands-on-Science by University of Minho (6, 14, 15)]. With these partnerships, scientists had the opportunity to share their research with teachers, to illustrate some experiments, and to collaborate in developing high-quality teaching programs. A continuous teacher training has previously been shown to positively affect student’s personal attitudes and academic performances (9, 16). For instance, the acquisition of scientific knowledge in health areas can play a crucial role in teaching adolescents to think critically and encourage healthy behaviors (16). The precocious awareness toward ecological problems can also help young students to develop environment-friendly behaviors (17). However, to make the most of the potential of these partnerships, scientists need to recognize the true needs and interests of secondary school teachers and of their students.

This work is based on the outcomes of an educational study implemented with science teachers (Biology, Chemistry, Geology, Physics and Geography) from Portuguese Basic and Secondary schools. This research aims to investigate the effectiveness of scientific lectures included in the “IV Cycle of Scientific Conferences 2015,” in improving teacher scientific knowledge. These cycles of conferences are organized regularly in an annual basis (it is in its fourth edition). In this cycle of conferences, several academic teachers/scientists from different fields are invited to perform lectures to Basic/Secondary school teachers’, by putting them abreast of the latest research in their respective fields. The “IV Cycle of Scientific Conferences 2015” was constituted by nine lectures lasting 1 h and 45 min followed by 15 min of discussion. It also included one field class (7 h) regarding the impact of mining, which was an important activity in Portuguese economy, in the last century, but also with severe environmental consequences. With the new perspectives provided by these lectures, science teachers can play a key role in updating students with high quality knowledge and skills, to inform about new careers/opportunities and foment scientific interest, which is the basis for solving several societal challenges.

**Figure 1:**
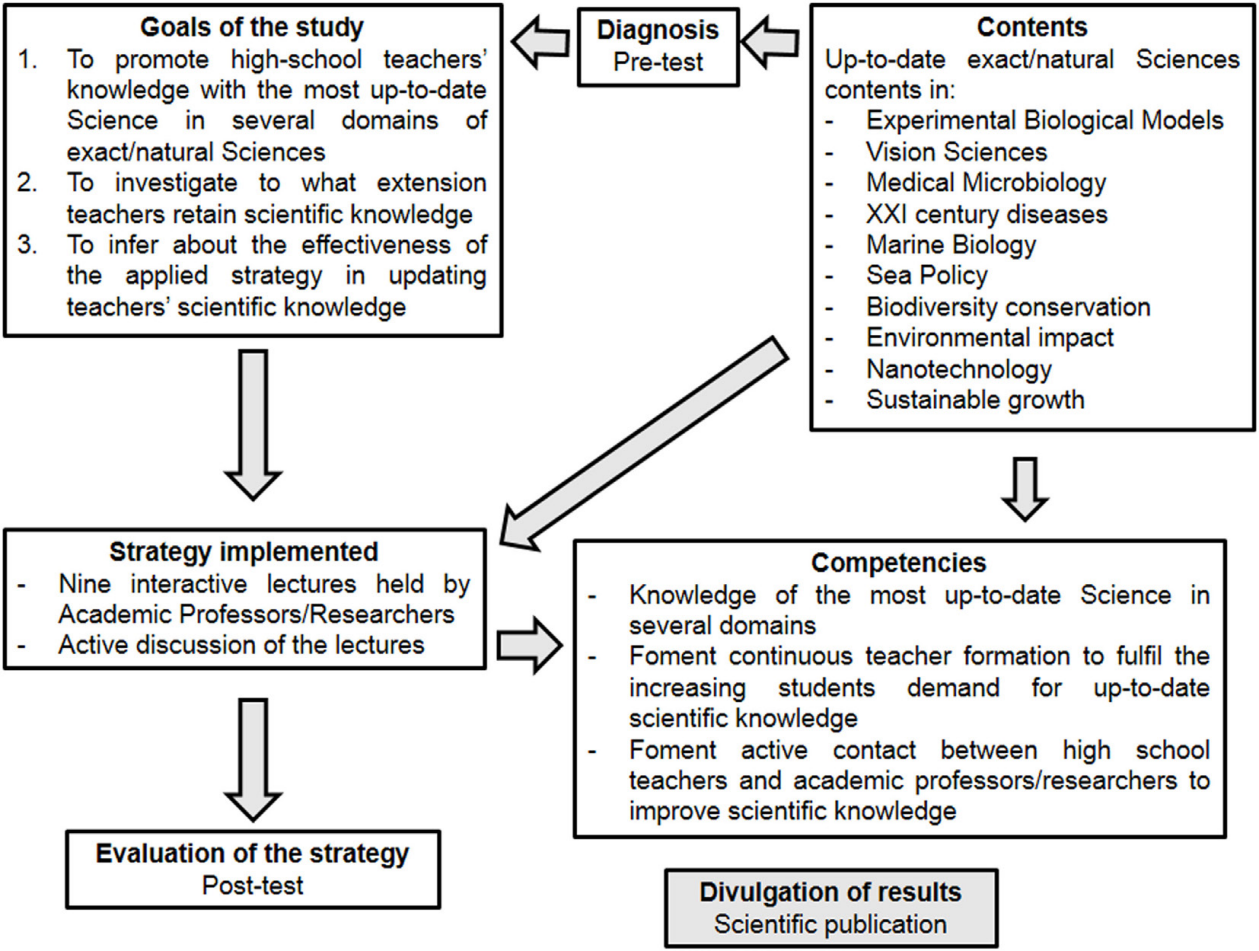
Outline of the implemented strategy of the current study including goals contents, expected competencies, and strategies applied.

**Figure 2:**
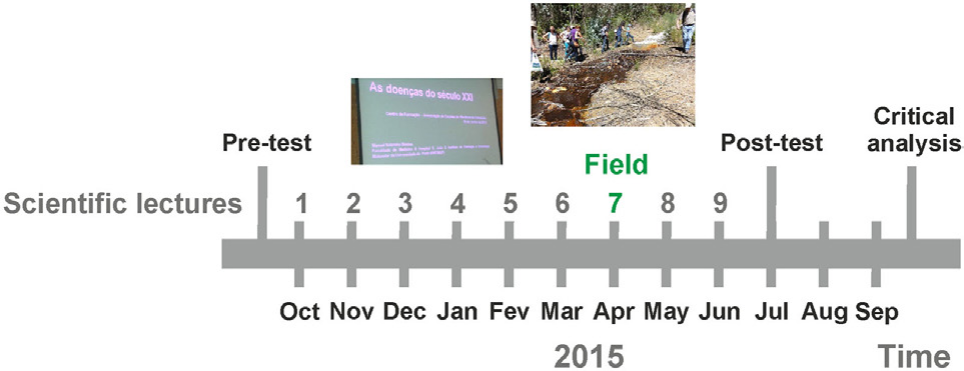
Timeline depicting the major events that took place during the “IV Cycle of Scientific conferences 2015.”

The goals of the current study were (i) to evaluate the knowledge of science teachers in several domains before the implementation of the scientific lectures, (ii) to investigate to what extent science teachers retain the knowledge, after the lectures and to infer about the effectiveness of the strategies applied, (iii) to open new perspectives to the teachers, and (iv) to promote reflection. A pre- and post-test questionnaire covering various topics of science was used to assess teachers’ knowledge. A pre-test (T0) was applied in October, before the first lecture and a post-test in July (T1), at the end of the lectures (Figures 1 and 2).

## Materials and Methods

### Study Method

#### Research Questions

The research questions addressed in this study aimed to confirm the following hypotheses:

(a) The knowledge of the teachers who attended this training activity is progressive along the study T0 versus T1;
(b) Teachers knowledge from the test group in T1 is considerably higher than the knowledge in the control group in T1.

### Participants

This study was developed between October 2014 and July 2015 and involved teachers who attended a training activity entitled “IV Cycle of Scientific Conferences 2015” held in V. N. Famalicão (test group, *n* = 35), and teachers who did not attend to this training activity (control group, *n* = 10). The teachers who attended to “IV Cycle of Scientific Conferences 2015” belong to six different schools of V. N. Famalicão County (D. Maria II, Camilo Castelo Branco, Júlio Brandão, D. Sancho I, Ribeirão, and Joane).

For the present work, we used a convenience sample, detailed in Table 1. Ethical approval for the study was obtained from the director of the Training Center (V. N. Famalicão). Informal verbal consent was obtained from all the participants. The participation was voluntary and anonymous. Participants were informed about the study, and it was assured that no participant would be identified. There were no refusals to the participation in the current study.

**Table 1:** Characterization of the test and control groups.

Class	No. of students	Age
**Test group**		
Teachers who attended to the “IV Cycle of Scientific Conferences 2015”	35	34–56
**Control group**		
Teachers from D. Maria School V.N. Famalicão	10	43–57

Total	45	34–57

### Brief Description of the Scientific Lectures

Announcements about this initiative were sent to all the schools of V. N. Famalicão County. To ensure quality, a well-equipped auditorium with audiovisual support was made available. Below, there is a brief description of the scientific lectures held during the “IV Cycle of Scientific Conferences 2015”:

(1) “From the clinic to the bench: how to develop and validate an animal model of osteoarthritis,” performed by Filipa Pinto Ribeiro (Ph.D., University of Minho, Braga, Portugal). The lecturer explained what is an experimental biological model. The most recent results of the lecturer’s research concerning the study of osteoarthritis, using the rat as experimental biological model, were shown. These topics are addressed in the program of the Biology/Geology discipline curricula, in the 10th and 11th grades of Portuguese secondary school, which has as guidelines the reinforcement of abstraction skills and experimentation. This content can also be explored by teachers under the module of diseases and biotechnology control, included in Biology discipline curricula from the 12th grade.
(2) “See without comprehension,” performed by Miguel Rocha (Optometrist, Póvoa de Varzim, Portugal). The lecturer highlighted about the main learning difficulties expressed by students due to vision problems in several capabilities such as visual acuity, binocular vision, accommodation, and eye movement. These capabilities, when poorly developed, can strongly affect the reading and learning processes. These contents can be explored by teachers of Physics and Chemistry of the eighth grade, which has a module about optical phenomena that characterize common vision defects and the type of lenses that can be used to correct them.
(3) “A fright called *Legionella*,” performed by Sofia Costa de Oliveira (Ph.D., University of Porto, Porto, Portugal). The lecturer talked about the discovery and the history of the *Legionella* bacteria responsible for an outbreak of pulmonary infection in 2014, in Vila Franca de Xira, Portugal. These contents can be explored by teachers in the module of diseases and biotechnology control included in the Biology discipline curricula of the 12th grade.
(4) “21th century diseases,” performed by Manuel Sobrinho Simões (MD, Ph.D., University of Porto, Porto, Portugal). The lecturer addressed the problem of civilizational and emerging diseases of the twenty-first century with a worldwide impact, such as obesity, diabetes, AIDS, tuberculosis, depression, neurodegenerative diseases, geriatric diseases, and cancer, which can be largely influenced by the environment/lifestyle even more than by genetic factors. These contents can be explored by teachers in the module about diseases and biotechnology control, included in the Biology discipline curricula of the 12th grade.
(5) “The Portuguese national strategy for the oceans 2013/2020,” performed by Sandra Paiva (Ph.D., University of Minho, Braga, Portugal) and “The health and the conservation of marine ecosystems,” performed by Jorge Santos (Ph.D., University of Minho, Braga, Portugal and Vigo University, Vigo, Spain). In a first part, Sandra Paiva addressed the new model for ocean and coastal areas development implemented in Portugal. In a second part, Jorge Santos highlighted the importance of marine species conservation in Portugal Continental developed under the framework of the LIFE + MarPro project. These topics are addressed in the Biology/Geology discipline curricula of the 10th and 11th grades, which has as guidelines the enhancement of biological diversity in its multisystem dimensions, structural and functional; the valorization of interdependence man–environment and the enhancement of biological evolution as a process that ensures biodiversity. The contents can also be explored by Biology teachers of the 12th grade in the module of natural resources and sustainability. These topics can also be discussed in the discipline of Geography, of the third cycle from Basic School, in the module of economic activities, such as agriculture and fisheries, and in the 10th and 11th grades from secondary school, under the modules of marine resources and environmental recovery in Portuguese and community environmental policy.
(6) “Diversity and activity of stream-dwelling decomposer fungi,” performed by Sofia Duarte (Ph.D., University of Minho, Braga, Portugal). In this lecture, the effects of anthropogenic stress (such as eutrophication and metal pollution) in the activity and diversity of stream-dwelling decomposer fungi were presented. These topics are addressed in the discipline of Biology/Geology of the 10th and 11th grades, which has as guidelines to relate the structure and function at all levels of biological organization and to assess the interactions of living systems with the environment, exchanging matter, and energy. These topics can be explored in the 12th grade, during Biology classes, in the module of natural resources and sustainability.
(7) “Monitoring and remediation of degraded mining areas,” lecture and field trip performed by Teresa Valente (Ph.D., University of Minho, Braga, Portugal). In this theoretical–practical session, the lecturer addressed the environmental impact associated with the quarrying/mining activity, which poses one of the most serious environmental impacts in Portugal and several other countries, particularly the contamination of river systems by acid drainage waters. In the practical session, the teachers analyzed the main determinants of acid drainage waters process generation, and characterized receptor systems of acid drainage waters by using physical–chemical and ecological indicators. The case study was an abandoned mine that is being rehabilitated, whose residues and effluents affect the Coura River, belonging to the Minho river basin. This basin is highly important for providing water for the North region of Portugal, particularly in the Minho region. The multidisciplinary approach used by Teresa Valente can be mimicked for planning a field class for secondary school students, since the topics addressed covered multiple contents of the Biology, Chemistry, and Geography disciplines curricula.
(8) “Applications and toxicity of nanomaterials: two sides of the coin,” performed by Fernanda Cássio (Ph.D., University of Minho, Braga, Portugal). The lecturer presented the potential of nanotechnology applications in several fields such as in medicine, pharmaceutical industry, agriculture, electronic, environment, textile industry, automobile industry, construction, and aeronautics, but also highlighted the negative impact that nanoparticles can pose to the environment and living species. These contents could be explored in Basic and Secondary school in the Chemistry, Biology, and Geography disciplines.
(9) “Green growth,” performed by Jorge Moreira da Silva (Engineer, Minister for Environment, Spatial Planning and Energy of Portugal). The Minister presented the “Commitment to Green Growth” that determines a green economic growth with a high national impact, but that also aims to increase the international visibility of Portugal as an upright example in what concerns environmental quality. The speaker reinforced the green growth as one of the pillars of the sustainable development model and highlighted the decisive role that teachers and young students can have in the implementation of environmental policies and as primordial agents of change to more sustainable behaviors. These contents are in line of the Natural Sciences disciplines curricula of 8th grade and of the 12th grade of Biology under the module of natural resources and sustainability, and in the line of the Geography discipline curricula of the third cycle of Basic school under the module of environment and society and major environmental challenges.

The strategies used by the lecturers were diversified; these were preferably based on active learning, which is in line with the guiding principles of the Bologna Declaration. The lecturers implemented several dynamic activities such as videos and PowerPoint projection with presentation/analysis/discussion of scientific results and informatics tools, in order to explore the contents covered. These tools are crucial to inspire and motivate the teachers, and to open their minds to new perspectives. The theoretical/practical lectures were structured around the themes focused in the pre-test.

### Data Collection and Analysis

The quantitative results of this study were obtained through two-time application of a pre-validated questionnaire during the training activity. The questionnaire was pre-validated with a sample of 20 subjects (teachers). A pre-test was applied in the first lecture (October, T0), and a post-test, after the latest lecture (June, T1) (Figure 2).

The questionnaire contained 19 questions covering the several topics addressed during the training activity. Questions were drafted by the authors of the current study. The questions are depicted in Tables 2 and 3, and covered the following topics: experimental biological models (Q1, Q2); vision sciences (Q3, Q4); medical microbiology (Q5, Q6); 21th century diseases (Q7); marine biology and species conservation (Q8, Q9, Q10, Q11); microbial ecology of streams (Q12, Q13); environmental impact of mining activities (Q14, Q15, Q16); and nanotechnology and nanoparticles (Q17, Q18, Q19).

**Table 2:** Performance in the pre- (T0) and post-test (T1) for the test group.

Questions	Correct answers (%)		Fisher’s exact test
	T0	T1	*P-*value
Q1. Before being brought to market, new drugs are first tested on: (select the correct options) (a) Plants (b) Eukaryotic microorganisms (c) Animals (d) Humans	3	3	0.7

Q2. What do you understand by experimental model of disease?	6	23	0.05

Q3. With regard to vision: (select the correct option) (a) The vision is born ready (b) The vision develops (c) The vision becomes more perfect with the time (d) All the options are correct	10	54	0.0001

Q4. For a good read is/are required (s) (select the correct option) (a) Eye movements (b) Good eyesight (c) Functional binocular vision (d) Accommodation with flexibility (e) All of the above options	63	94	0.002

Q5. Indicate where you can find the bacteria *Legionella*	12	49	0.001

Q6. Suggest two preventive measures to combat *Legionella*	36	57	0.07

Q7. Indicate the two major diseases of twenty-first century and their causes	23	40	0.1

Q8. Oceans are: (select the correct option) (a) The largest and least exploited part of the planet Earth; less than 5% of the oceans are known (b) The largest and most exploited part of the planet Earth; over 75% of the oceans are known (c) The largest and most exploited part of the Earth; approximately 50% of the oceans are known (d) None of the options is correct	77	91	0.1

Q9. Indicate four competitive advantages of Portugal in relation to other European countries with regard to the sea	33	40	0.4

Q10. The Network Natura 2000 is one of the main tools of the EU’s policy for the protection of nature and biodiversity. The project LIFE MarPro supports the extension of the Network Natura 2000 for ocean areas of mainland Portugal in order to conserve marine species protected in mainland Portugal. These species include: (select the correct option) (a) Boto (cetacean) (b) Roaz-corvineiro (cetacean) (c) Pardela-balear (bird) (d) All the species listed above are included in the project’s conservation program LIFE MarPro (e) None of the options is correct	57	89	0.004

Q11. In addition to the conservation of protected marine species in continental Portugal, select other aims of the project LIFE MarPro: (select the correct option) (a) Encourage the capture of protected species (b) Contribute to reduce conflicts between fisheries and cetaceans/seabirds (c) Reduce the communication links between users of the marine environment (d) Options b and c (e) All options are correct (f) None of the options is correct	73	74	0.6

Q12. What is the main process by which organisms (e.g., microorganisms, invertebrates) that inhabit the small order streams get nutrients and energy? (select the correct option) (a) Decomposition of litter from the riparian vegetation (b) Photosynthesis (c) Both processes are important (d) None of the processes is important	79	88	0.3

Q13. Aquatic fungi are the dominant decomposer microorganisms of litter that falls into streams due to: (select the correct option) (a) The ability to remain active at low temperatures (b) Capacity of production of a wide range of extracellular enzymes responsible for the degradation of complex polymers such as cellulose, hemicellulose, and pectins (c) Capacity of production of a large amount of spores (reproductive structures) with tetraradiate forms (d) All options are correct (e) None of the options is correct	18	57	0.001

Q14. Mention the main activities that generate environmental impact resulting from the mining industry: (select the correct option) (a) Prospection (b) Disassemble—exploitation of the mineral deposit (c) Abandonment of reactive sterile in heaps (d) Transport of minerals and reactive sterile (e) Concentration of minerals from the extracted material	0	14	0.04

Q15. Acid drainage waters are: (select the correct option) (a) Acidic aqueous solutions resulting from mineral acidic treatment (b) Effluents resulting from mining sulfur farm for the production of sulfuric acid (c) Aqueous solutions resulting from the oxidative dissolution of sulfides (d) Mining effluents produced in abandoned mines (e) Contaminated aqueous solutions generated by the weathering of silicates and carbonates	7	80	<0.0001

Q16. Which of the following are examples of biological indicators of mine contamination: (select the correct option) (a) *Euglena mutabilis* and *Klebsormidium* sp. (b) *Euglena granulata* and *Klebsormidium* sp. (c) *Euglena granulata* and *Euglena viridis* (d) *Euglena granulata* and *Euglena gracilis* (e) *Pinularia aljustrelica* and *Euglena viridis*	7	80	0.0002

Q17. Regarding the size of the nanoparticles: (select the correct option) (a) They are between 1 and 5,000 nm (b) They are between 1 and 2,500 nm (c) They are between 1 and 1,000 nm (d) They are between 1 and 500 nm	17	71	<0.0001

Q18. Indicate three nanotechnology applications	27	77	0.0001

Q19. What is INL?	10	49	0.0001

The qualitative results were obtained through the conception of a report by the teachers who attended the lectures. Completion of this report is mandatory by the Training Center for the teachers to obtain a certificate that worth one credit. Every year, in the Portuguese National Teachers system, all the Basic and Secondary teachers have to attend 25 h of training to actualize knowledge, which corresponds to one (1) credit. In this report, the teachers under training had to make a general critical analysis of all the lectures and a more detailed analysis about one of the lectures by mentioning if their initial expectation concerning the conferences was attained, and if the contents can be applied on their regular and future pedagogic practice.

Data obtained in two different time points [T0—pre-test versus T1—post-test were analyzed using Statistica software for Windows, version 8.0 (Statsoft, Inc., Tulsa, OK, USA)]. Associations between variables were tested with Fisher’s exact tests with significance set at *P* < 0.05.

## Results

### Quantitative Results

The questions are depicted in Tables 2 and 3 and covered the topics detailed above. The response rate was 100% among the 35 teachers asked to participate. The characteristics of the study and control populations are shown in Table 1. Data presented in Tables 2 and 3 show the knowledge of the teachers regarding the several topics covered by the scientific lectures, before (pre-test, T0) and after (post-test, T1) these being implemented, for the test and control groups, respectively. For all questions (with the exception of Q1), there was an increase in the % of correct answers among the test group (Table 2). Most of these increases were significant in all covered areas such as: vision sciences (Q3, Q4), medical microbiology (Q5), marine biology and species conservation (Q10), microbial ecology of streams (Q13), and environmental impact of mining activities (Q14, Q15, Q16) and nanotechnology and nanoparticles (Q17, Q18, Q19) (Table 2, Fisher’s exact tests, *P* < 0.0001–0.04).

There were seven questions for which no significant differences were found between the answers in the pre- and post-tests (Table 2, Fisher’s exact tests, *P* = 0.05–0.7), but for which a good percentage of correct answers were obtained immediately in the pre-test. These were related with the preventive measures to combat *Legionella* (Q6), the major diseases of the 21th century (Q7), Portuguese sea policy (Q8, Q9), protected marine species in Portugal (Q11), and general ecological processes occurring in aquatic ecosystems (Q12), which are topics that are either covered in regular Science classes or that had/has high impact in Portuguese environment and socioeconomics. Exceptionally for Q1, which was related with the models used to test new drugs before being introduced in the market, the % of correct answers was too low (3%) either in T0 or T1.

In general, for the control group, which was constituted by teachers who did not attend to the scientific lectures, no significant differences were found between the answers in T0 and T1 (Table 3, Fisher’s exact tests, *P* = 0.1–0.8). Exceptionally, for Q1 and Q5, which were related with the sources of *Legionella* bacteria in the environment and experimental biological models, respectively, a significant increase in the % of correct answers was found between T0 and T1 (Table 3, Fisher’s exact tests, *P* = 0.03 and *P* = 0.003, respectively). This increase in the % of correct answers may be related with knowledge acquisition from other sources, such as the media. The sources where *Legionella* bacteria can be found in the environment were strongly reported in the Portuguese media due to an epidemic burst in Portugal, which occurred in the winter of 2014.

**Table 3:** Performance in the pre- (T0) and post-test (T1) for the control group.

Questions	Correct answers (%)		Fisher’s exact test
	T0	T1	*P-*value
Q1. Before being brought to market, new drugs are first tested on: (select the correct options) (a) Plants (b) Eukaryotic microorganisms (c) Animals (d) Humans	10	80	0.003

Q2. What do you understand by experimental model of disease?	0	20	0.2

Q3. With regard to vision: (select the correct option) (a) The vision is born ready (b) The vision develops (c) The vision becomes more perfect with the time (d) All the options are correct	0	0	–^a^

Q4. For a good read is/are required (s) (select the correct option) (a) Eye movements (b) Good eyesight (c) Functional binocular vision (d) Accommodation with flexibility (e) All of the above options	80	50	0.2

Q5. Indicate where you can find the bacteria *Legionella*	30	80	0.03

Q6. Suggest two preventive measures to combat *Legionella*	0	10	0.5

Q7. Indicate the two major diseases of twenty-first century and their causes	30	50	0.3

Q8. Oceans are: (select the correct option) (a) The largest and least exploited part of the planet Earth; less than 5% of the oceans are known (b) The largest and most exploited part of the planet Earth; over 75% of the oceans are known (c) The largest and most exploited part of the Earth; approximately 50% of the oceans are known (d) None of the options is correct	100	80	0.2

Q9. Indicate four competitive advantages of Portugal in relation to other European countries with regard to the sea	30	40	0.5

Q10. The Network Natura 2000 is one of the main tools of the EU’s policy for the protection of nature and biodiversity. The project LIFE MarPro supports the extension of the Network Natura 2000 for ocean areas of mainland Portugal in order to conserve marine species protected in mainland Portugal. These species include: (select the correct option) (a) Boto (cetacean) (b) Roaz-corvineiro (cetacean) (c) Pardela-balear (bird) (d) All the species listed above are included in the project’s conservation program LIFE MarPro (e) None of the options is correct	80	60	0.3

Q11. In addition to the conservation of protected marine species in continental Portugal select other aims of the project LIFE MarPro: (select the correct option) (a) Encourage the capture of protected species (b) Contribute to reduce conflicts between fisheries and cetaceans/seabirds (c) Reduce the communication links between users of the marine environment (d) Options b and c (e) All options are correct (f) None of the options is correct	70	70	0.7

Q12. What is the main process by which organisms (e.g., microorganisms, invertebrates) that inhabit the small order streams get nutrients and energy? (select the correct option) (a) Decomposition of litter from the riparian vegetation (b) Photosynthesis (c) Both processes are important (d) None of the processes is important	30	50	0.3

Q13. Aquatic fungi are the dominant decomposer microorganisms of litter that falls into streams due to: (select the correct option) (a) The ability to remain active at low temperatures (b) Capacity of production of a wide range of extracellular enzymes responsible for the degradation of complex polymers such as cellulose, hemicellulose, and pectins (c) Capacity of production of a large amount of spores (reproductive structures) with tetraradiate forms (d) All options are correct (e) None of the options is correct	30	40	0.5

Q14. Mention the main activities that generate environmental impact resulting from the mining industry: (select the correct option) (a) Prospection (b) Disassemble—exploitation of the mineral deposit (c) Abandonment of reactive sterile in heaps (d) Transport of minerals and reactive sterile (e) Concentration of minerals from the extracted material	0	10	0.5

Q15. Acid drainage waters are: (select the correct option) (a) Acidic aqueous solutions resulting from mineral acidic treatment (b) Effluents resulting from mining sulfur farm for the production of sulfuric acid (c) Aqueous solutions resulting from the oxidative dissolution of sulfides (d) Mining effluents produced in abandoned mines (e) Contaminated aqueous solutions generated by the weathering of silicates and carbonates	20	0	0.2

Q16. Which of the following are examples of biological indicators of mine contamination: (select the correct option) (a) *Euglena mutabilis* and *Klebsormidium* sp. (b) *Euglena granulata* and *Klebsormidium* sp. (c) *Euglena granulata* and *Euglena viridis* (d) *Euglena granulata* and *Euglena gracilis* (e) *Pinularia aljustrelica* and *Euglena viridis*	10	10	0.8

Q17. Regarding the size of the nanoparticles: (select the correct option) (a) They are between 1 and 5,000 nm (b) They are between 1 and 2,500 nm (c) They are between 1 and 1,000 nm (d) They are between 1 and 500 nm	20	30	0.5

Q18. Indicate three nanotechnology applications	10	40	0.1

Q19. What is INL?	20	40	0.3

*^a^Not possible to apply Fisher’s exact test*.

### Qualitative Results

After the analysis of the reports, the teachers under training clearly demonstrated a high interest in all the subjects covered during the “IV Cycle of Scientific Conferences 2015,” since all lectures were selected at least once during their critical reflection, with the exception of the lecture related with experimental biological models. The teachers also mentioned in their reports that all expectations concerning these conferences were successfully attained and even exceeded. The teachers under training declared that one of the strongest points of these conferences were the high scientific quality and innovative nature transmitted by the researchers in a relaxed way, and they reported that most of the acquired knowledge can be easily applied during their regular pedagogical practice. The teachers also pointed as one of the strongest aspects that all the contents covered are integrated in the National Portuguese Basic/Secondary teaching programs of Natural Sciences, Biology, Physics, Chemistry, and Geography. For the critical analysis that was requested about one of the lectures, to the teachers under training, the selection was the following: “21th century diseases”: 8; “A fright called *Legionella*”: 7; “The Portuguese national strategy for the ocean 2013/2020” and “The health and the conservation of marine ecosystems”: 7; “See without comprehension”: 5; “Green growth”: 4; “Diversity and activity of stream-dwelling decomposer fungi”: 2; “Monitoring and remediation of degraded mining areas”: 1 and “Applications and toxicity of nanomaterials: two sides of the coin”: 1. All the reports had a classification of “Excellent” (between 9 and 10 points).

### Observation of the Teachers

During the lectures, teachers have shown a great interest in all the topics addressed. The attendance was 100% to all the lectures. The invited academic teachers/scientists promoted a highly interactive discussion by asking questions and pointing out interesting issues concerning the several topics addressed and making connection with real cases. The used approaches encouraged and motivated the teachers to reflect and to acquire efficiently the transmitted knowledge. The teachers participated actively in all the activities proposed, including the field trip.

## Discussion

Scientific education plays a major role in sustaining and improving scientific literacy of the wider population and in encouraging and motivating young students to become interested in science and even to embrace a scientific career (18). Due to their close proximity, teachers can play a crucial role in students’ scientific literacy acquisition and motivation to respond to ongoing and future economic and societal challenges. Adult education has been proven to positively impact adults’ lives and help them to live more successfully, by increasing competencies that help citizens to solve several personal and community issues (19). Although all adults are encouraged to invest in lifelong learning (see text footnote 1), for teachers, this should be mandatory due to the crucial role that they can play in societies in transmitting up-to-date knowledge. Good teachers must be enthusiastic, motivated, proactive, confident, flexible, and competent. In particular, science teachers should be able to formulate adequate questions, test ideas, collect and analyze data, support arguments and collaborate with peers, to actualize scientific knowledge and learn how to use it. For instance, the Portuguese Ministry of Education is nowadays highlighting the need to increase scientific literacy, which is explicit in the “National Curriculum for Basic Education-Essential Skills,” Currículo Nacional do Ensino Básico (20). Based on these assumptions, a cycle of scientific conferences has been designed in the last 4 years to provide scientific literacy to Basic/Secondary school teachers of sciences, from V. N. Famalicão County (Portugal). These conferences were held by academic teachers/scientists from various fields of sciences. However, although this strategy was already implemented three times in the past, no assessment has been conducted yet to date to assess its effectiveness. In fact, very little research has been conducted to demonstrate the effectiveness of training interventions. Previous evidence suggests a positive impact of teacher training programs in terms of quality of communication, teacher-student relationships and strategies applied during regular classes (21, 22). However, most studies have relied on indirect measures, such as learner satisfaction surveys or self-assessment by participants (23–25).

For the reasons pointed above, we have conducted the current study to quantify the importance of a training action engaging Basic/Secondary school teachers of sciences. The expectations by the teachers were very high, regarding the invited lecturers and the methodologies to be applied, which may have also motivated a fast acquisition of the transmitted knowledge. The lectures also embraced very different topics, which may have had also a positive impact on the knowledge acquisition. According to previous studies, a diversification of lectures is crucial to create an environment conducive to learning (26, 27). All the lectures addressed authentic Portuguese and European concerns related mainly with health and environmental issues. Health literacy is critical to allow citizens to exert greater control over their health and to promote healthy behaviors (28), while environmental literacy is crucial to deal with the global change that the world is experiencing nowadays (29).

In the current study, for the test group (that attended the training activity), an increase of the percentage of correct answers was observed in almost all questions in T1 (after the training activity), compared with T0 (before the training activity) (Table 2), supporting our first hypothesis. On the other hand, for the control group (that did not attend to the training action), no significant differences were found between T0 and T1 for most of the questions (Table 3), supporting our second hypothesis.

The high quality of the lectures provided, as well as the up-to-date topics addressed in both areas—health and environment—have definitely contributed for the high motivation and improved results among the individuals of the test group in T1. For instance, in 2014, a *Legionella* outbreak struck occurred in Portugal in 2014, which was one of the topics approached during the lectures related with health. The high expectation also clearly influenced teachers’ motivation in knowledge acquisition about health topics. The lecture about the 21th century diseases was held by a much known Portuguese Scientist that beyond of being an excellent Researcher and Doctor, revealed to be an excellent speaker. The high interest generated by this lecture was mirrored in the high percentage of correct answers in T1 and also by the high number of choices as topic for conducting the critical analysis. The connection of the theoretical knowledge with practical examples of everyday life, such as those provided in the lecture about sciences vision (e.g., how vision can strongly influence the success of the teaching-learning process), may have also contributed for the significant increase of correct answers in T1.

In our study, an increase in the number of correct answers in T1 for the test group was also observed for most of the questions related with the environment (Table 2). One of the topics discussed in the lectures was about the potential of the ocean/coastal areas to improve the competitiveness of the Portuguese maritime economy. In addition, emphasis was put on the importance of marine species conservation in Portugal, due to the valuable ecosystem services that they provide. The high success of this lecture was both demonstrated by the high % of correct answers in T1, as well as by the number of choices as topic for conducting the critical analysis, among the test group. Numerous studies have previously shown the importance of public participation in environmental conservation initiatives (30, 31). However, before an individual can intentionally act on a particular environmental issue, it must be first conscious of the existence of that problem (32). Thus, knowledge involving biodiversity conservation-related issues would not only be important to teachers but also to the population in general.

Topics that are more similar to those that teachers need to address in their regular classes may also improve significantly knowledge acquisition. In the lecture about the diversity and activity of stream-dwelling decomposer fungi in freshwaters, and effects of anthropogenic effects, the lecturer explained some methodologies that can be used for collecting aquatic fungi from streams suffering from different environmental perturbations. In fact, two of the teachers mentioned in their critical analyses that, after this lecture, they had applied some of these methodologies during experimental classes, which were easily integrated under the theme of the impact of pollution on aquatic ecosystems taught in Basic and Secondary schools in Natural Sciences or Biology disciplines.

Some other topics were new to the majority of the teachers, such as those addressed in the lectures about monitoring and remediation of degraded mining areas and the applications and toxicity of nanomaterials, which were supported by the feeble results obtained in T0. However, the former lecture was reinforced with fieldwork, which seemed to highly stimulate the curiosity of the teachers for the topics addressed. Data from the literature corroborate the benefits of fieldwork implementation, and the importance of active participation in collaborative work compared with traditional lecturing (33). The high percentage of correct answers in T1 in our study corroborates the benefits that fieldwork can have in the acquisition of new information. Active learning has been shown to yield better outcomes than traditional learning (34, 35). In addition, during active learning, teachers can acquire important skills such as teamwork, design of scientific experiments, as well as analysis and interpretation of data, which can greatly improve their teaching methodologies.

Although the last lecture was not quantitatively evaluated (the confirmation of this talk was made very late), the raised interest is worth mentioning since four teachers chose this topic—green growth—for performing the critical analysis. The politics of “green growth” is a major concern nowadays in all countries of the European Union, since the development of environmental protective measures is increasing to delay the effects of global change. A great discussion was generated among the lecturer (Minister of Environment, Spatial Planning, and Energy of Portugal) and the teachers, because some of the measures implemented in Portugal are already having repercussions in their day-by-day lives. Thus, addressing present-day topics may also significantly improve teachers’ motivation to actively participate in the discussions and foment a greater knowledge acquisition.

However, not all the knowledge on the topics addressed during the lectures were efficiently acquired. In the current study, most of the teachers were not able to explain what is an experimental model of disease, after attending the lecture on this topic. In addition, most of them claimed that new drugs to be introduced in the market should be first tested in plants and humans, rather than being first tested in eukaryotic microorganisms or animals. We believe that the difficulty level of this lecture was high, since it was based on very fundamental science. In this conference, the lecturer presented a specific case where osteoarthritis was induced in rats and the therapeutic effects of several drugs monitored. These results revealed that the teachers under training were not capable of establishing the link between the knowledge transmitted from specific cases to general biology models (eukaryotic microorganisms, animals). This was also reflected in their critical analysis of the cycle of conferences since none of the teachers selected this lecture. Therefore, we may conclude that science communication should embrace more general contents of science and how science can be used to tackle societal challenges.

The teachers under training felt that these scientific lectures were very appropriate and have positively impacted their current knowledge, as well as their teaching methods. The current analysis will be extremely valuable for revising, improving, and reinforcing some of the contents addressed in future actions of this kind. A recent investigation showed the importance of continuous teacher development as a major factor that have a direct impact upon students’ academic performance (9). The high relevance of teacher training by the scientific community was earlier demonstrated as highly valuable in scientific knowledge actualization, experimental skills acquisition, and familiarization with the most recent techniques and protocols that can be applied in regular classes of science teaching (10). Therefore, links with the scientific community are crucial to make science teachers aware of the most recent findings of science and a major concern of the Educational Ministry during national curricula planning.

Other suggestions that may improve teacher scientific knowledge are: (i) to establish point collaborations with scientists from universities (the current training highly promoted this contact), (ii) to have access to specific training conducted by the universities (e.g., the teachers should be able to enroll in specific disciplines for which they feel a need for updating or training), and (iii) to create training teachers’ web pages as found in other countries of the European Union and United States (e.g., Corner/Galileo Teacher Training Program). Science coffee shops are also becoming more common and include several science events for adults that teachers can attend to be aware of most recent scientific developments.^2^

Concluding, our four goals presented earlier have been mostly achieved:

(i) The knowledge of the teachers in the several scientific domains in the first evaluation moment, T0, was weak, in particular, in the newest topics, to moderate, for more current topics;
(ii) The significant increase in the % of correct answers between T0, and the last evaluation moment, T1, among the individuals of the test group were quite satisfactory highlighting that the teachers were able to retain the acquired knowledge efficiently, at least for a short period of time (ca. 9 months). In addition, the greater % of correct answers compared with the control group, in T1, also reinforces the effectiveness of the strategies applied;
(iii) During their critical analysis, some of the teachers reported that they have used in their regular classes some of the methodologies that were transmitted during the scientific lectures, opening new perspectives to their teaching methods;
(iv) The teachers under training mentioned: “This kind of teaching is art and science”; “The subjects were very interesting and the speakers excellent”; “The lectures will allow to develop new approaches concerning the science curricula”; “My expectations were exceeded, it was a very rewarding and enriching experience from a personal and professional point of view,” promoting a critical reflection about the implemented activities and how they may change their teaching methods in a near future.

Although we have used a convenience sample, we should expect similar results if the same methodology is applied to teachers from different schools all over the country, since the curricula of science disciplines are the same. However, even so, our conclusions should be taken cautiously due to numerous extrinsic factors that can affect individual knowledge acquisition (e.g., pre-existent ideas). Thus, we suggest that further evaluations should be conducted by using larger samples, in order to check if the same findings are applied to the general population of our targeted audience. The generation of networks of teachers reporting their positive experiences, in science education, would be mandatory to “spread the word” and motivate the organization of more events of this kind in all the country.

## Informed Consent

Informed consent was obtained from all individual participants included in the study.

## Ethics Statement

This study was approved by the Director of the Training Center Camilo Castelo Branco. Teacher participation was anonymous and voluntary. The aims of this study were explained to the teachers previously, during a meeting, where the opportunity to make questions regarding the study was provided and where verbal authorization was requested. It was not formally asked for a participant written consent, because all participants voluntarily participated on this study and data were anonymously analyzed.

## Author Contributions

M-MA and SD designed the study, collected, analyzed the data, and wrote the paper.

## Conflict of Interest Statement

The author’s declare that the research was conducted in the absence of any commercial or financial relationships that could be construed as a potential conflict of interest.

## Footnotes

^1^http://www.aaace.org.

^2^http://www.pbs.org/wgbh/nova/blogs/education/wp content/uploads/sites/3/2016/02/NH-Science-cafe.jpg.
